# Viscoelastic Hemostatic Assays: Moving from the Laboratory to the Site of Care—A Review of Established and Emerging Technologies

**DOI:** 10.3390/diagnostics10020118

**Published:** 2020-02-21

**Authors:** Jan Hartmann, Matthew Murphy, Joao D. Dias

**Affiliations:** 1 Haemonetics Corporation, Boston, MA 02110, USA; MMurphy@Haemonetics.com; 2 Haemonetics SA, Signy CH, 1274 Signy-Centre, Switzerland; joao.dias@haemonetics.com

**Keywords:** blood, coagulation, hemostasis, point of care, ROTEM, TEG, thromboelastography, VHA, viscoelastic testing

## Abstract

Viscoelastic-based techniques to evaluate whole blood hemostasis have advanced substantially since they were first developed over 70 years ago but are still based upon the techniques first described by Dr. Hellmut Hartert in 1948. Today, the use of thromboelastography, the method of testing viscoelastic properties of blood coagulation, has moved out of the research laboratory and is now more widespread, used commonly during surgery, in emergency departments, intensive care units, and in labor wards. Thromboelastography is currently a rapidly growing field of technological advancement and is attracting significant investment. This review will first describe the history of the viscoelastic testing and the established first-generation devices, which were developed for use within the laboratory. This review will then describe the next-generation hemostasis monitoring devices, which were developed for use at the site of care for an expanding range of clinical applications. This review will then move on to experimental technologies, which promise to make viscoelastic testing more readily available in a wider range of clinical environments in the endeavor to improve patient care.

## 1. Introduction

The use of techniques to evaluate hemostasis utilizing the viscoelastic properties of whole blood have been reported since the 1940s. The technology and clinical applications of viscoelastic hemostatic assays grew slowly over the next 40 years. In parallel, the use of today’s standard hemostatic assays (prothrombin time, activated partial thromboplastin, international normalized ratio, etc.) became common. However, these assays only shed light on a small part of the overall hemostasis process. In the 1980s, the use of thromboelastography became more widespread during high blood loss procedures, such as cardiac surgery and liver transplantation [1,2], and in the management of major bleeding [3]. More recently, instrumentation quality and ease of use has improved dramatically. Viscoelastic hemostatic monitoring is now a rapidly growing field, drawing significant attention and investment. The current technological landscape has a host of new technologies being developed to improve the capability of the instrumentation and bring it closer to the site of care.

## 2. What Is Viscoelastic Testing?

**Figure 1:**
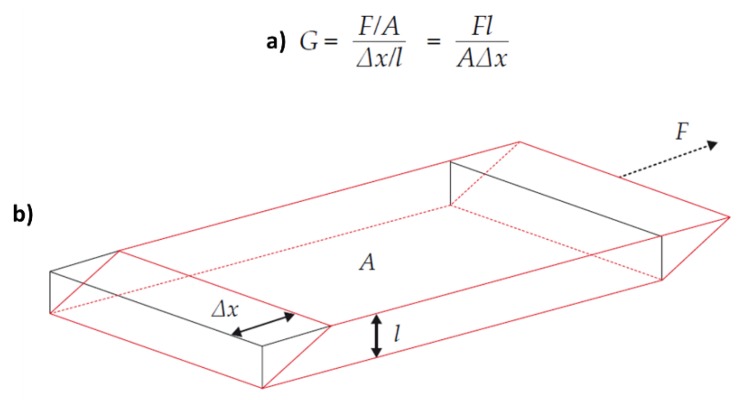
Shear elastic modulus. (**a**) Mathematical formula to express shear modulus; (**b**) Schematic representation of the shear principle. G = shear modulus; F = force; A = area; F/A = shear stress; Δx = transverse displacement; l = initial length. Reproduced with permission from [4].

Viscoelasticity is the characteristic of a material that behaves in both a viscous (permanent deformation) and elastic (temporary deformation) manner. Prior to clotting, whole blood is a purely viscous material and the response to shear stress will be permanent deformation. Once the shear stress is removed, the sample will not return to its original shape. As a blood sample begins to clot, the fluid becomes less viscous and more elastic in nature. The deformation induced by the shear stress becomes temporary as the fluid tries to return to its original shape. The primary measurement during viscoelastic hemostatic monitoring is the observation of the transition from a viscous to elastic state, and the measurement of the shear elastic modulus: a measure of the amount of force required to shear a material (also known as shear stiffness). The elastic modulus is defined as the ratio of shear stress to shear strain (Figure 1).

## 3. History of Viscoelastic Testing

**Figure 2:**
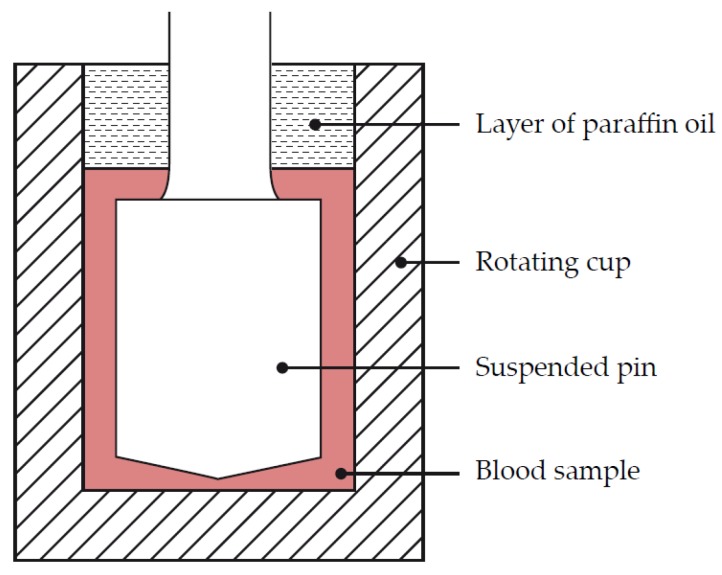
Dr. Hartert’s cup and pin mechanism. Schematic drawing representing parts of the thromboelastograph that were in direct contact with the blood sample. The rotating cup was approximately 8 x 12 mm and made from stainless steel, the surface of which prevented detachment of the blood clot during cup rotation. The blood sample was covered by a layer of paraffin oil to prevent evaporation of the sample. Reproduced with permission from [4].

Viscoelastic hemostatic monitoring was first described in 1948 by Dr. Hellmut Hartert in Germany [5,6]. Dr. Hartert wanted a mechanism to quantify the dynamics of blood clot formation. He developed a mechanism that consisted of a cup with a concentric pin suspended within (Figure 2). The pin was suspended with a thin steel wire with a diameter of 0.2 mm, which acted as a torsional spring.

**Figure 3:**
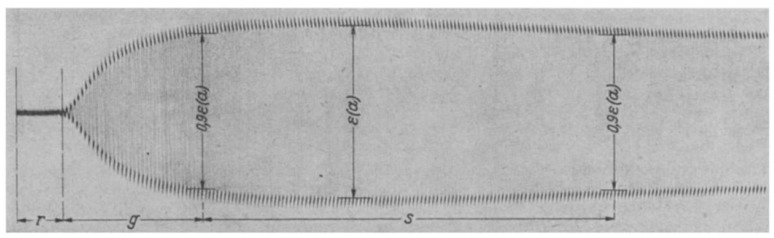
Output from Dr Hartert’s cup and pin. Representation of the output from the entire cycle of the cup and pin system. The R period was described as the reaction time, g as the growth of the clot and s as the stable period clot strength. The amplitude of the waveform is proportional to the shear modulus of the clot within its elastic region and is analogous to clot strength. Reproduced with permission from [5].

**Figure 4:**
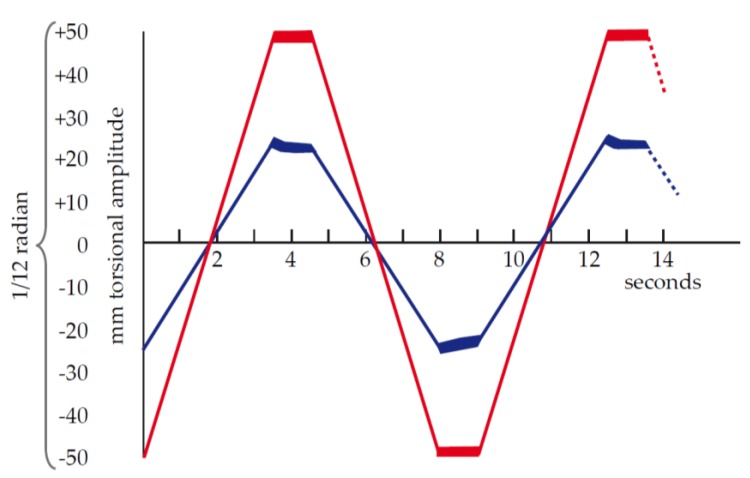
Chart representing the movement of Dr. Hartert’s cup and pin after clotting of the blood sample. The red line represents the displacement of the cup and the blue line represents the displacement of the pin. The units on the x-axis represent the extent of the illuminated section of film by the rotating mirror. The units of mm amplitude on the y-axis remain today in many viscoelastic hemostatic assay systems. The units of mm to express a clot strength has been the source of confusion in this space. Reproduced with permission from [4].

To perform a test, an activated sample of blood is placed in the cup and the pin lowered into place. The cup rotates in each direction by 1/24 radian, or 1/12 radian total rotation. The rotation occurs slowly, taking 3.5 s for one direction. The cup then comes to a stop for 1 s and then moves back in the other direction at the same speed. An entire “cycle” takes 9 s (two motion periods and two stationary periods). As the cup rotates with a viscous material (whole blood), the pin does not move. The shear between the rotating cup and stationary pin results in a permanent shear deformation of the blood. As the clot forms and grows in strength, the fluid within the cup begins its transition from a viscous to an elastic state. Energy is stored within the elasticity of the clot and the clot will try to return to its original shape, exerting a force on the pin, which causes the pin to rotate on its axis. The small rotations of the pin are transmitted to a film via a mirror coupled to the pin, which is illuminated by a slit lamp (Figure 3). The movement of the cup and pin after clotting is represented as a graphical chart in Figure 4.

Adoption of thromboelastography grew slowly following Dr. Hartert’s initial work, limited primarily to research laboratories. It began to gain some momentum in the 1980s, particularly in high blood loss procedures such as liver transplantation [1] and cardiac surgery [7,8]. Two similar technologies were initially developed, Thromboelastography (Thrombelastograph^®^ [TEG^®^] Hemostasis Analyzer) by Haemoscope, and Thromboelastometry (ROTEM^®^) by Tem International GmbH. These two technologies defined the first 30 years of clinically adopted viscoelastic hemostatic assays. Both technologies were enhanced by the utilization of different assays to gain visibility on different aspects of clot formation. The assays and associated reagents have been developed to investigate different coagulation pathways, the contributions of platelets and fibrinogen to clot strength, and the effect of clot lysis.

As the use of viscoelastic hemostatic assays has grown into more applications, its clinical value has increased. This has attracted significant academic interest. As such, next-generation technologies are rapidly being developed. The remainder of this review will focus on the first-generation technologies that have pioneered this field, the next-generation devices that have more recently gained regulatory approval, and the experimental technologies on the horizon.

## 4. First-Generation Devices

### 4.1. TEG^®^ 5000 (Haemoscope, Haemonetics)

The TEG^®^ 5000 system was the first VHA device available, originally developed by Haemoscope and then acquired by Haemonetics. The system has a long clinical history and has been widely published in the literature [9,10,11]. The mechanism of the TEG^®^ 5000 is very similar to what was originally developed by Dr. Hartert.

**Figure 5:**
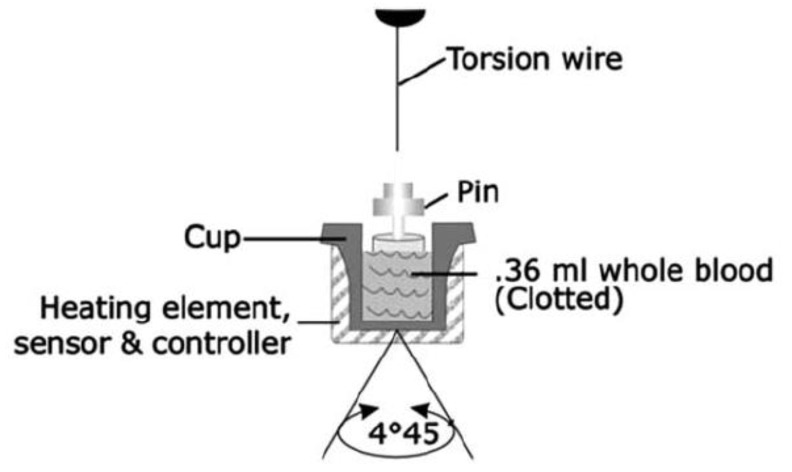
Schematic representation of the TEG^®^ 5000 system. Thromboelastography conducted with the TEG^®^ 5000 system uses approximately 0.36 mL of blood, which is placed into a cylindrical cup at 37 °C. A pin on a torsion wire is suspended in the blood, and the cup rotates in alternating directions (rotation angle 4°45’, cycle duration 10 s) to simulate venous flow. At the onset of each measurement, there is no torque between the cup and the pin, and the machine provides a reading of zero. As clotting occurs, fibrin fibers formed between the pin and the cup create a rotational force on the pin, which is measured via a torsion wire and an electromagnetic transducer; the readout line diverges from the baseline until it reaches a maximum value (maximum clot strength). With the onset of clot lysis, the readout converges back towards baseline. Reproduced with permission from Haemonetics [11].

**Figure 6:**
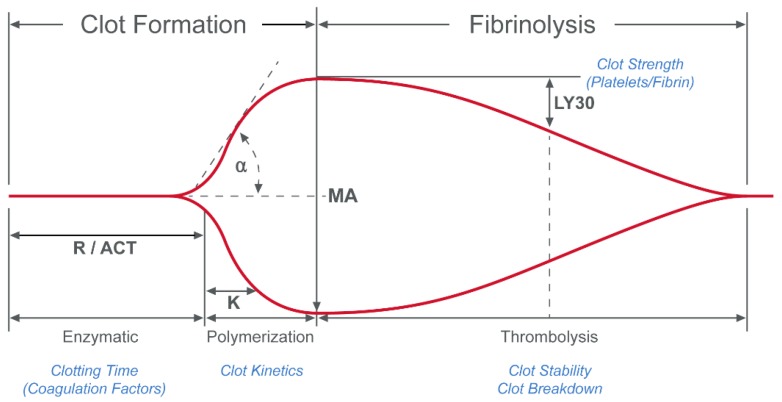
Plot of the total deflection of the pin from the TEG^®^ 5000 system. The primary values that are derived from the resulting waveform are reaction time (R, or activated clotting time [ACT]), maximum amplitude (MA) and lysis at 30 minutes (LY30). R represents the time to the beginning of clot formation. MA is the maximum clot strength achieved. LY30 quantifies the reduction of clot strength, or the lysis (in the 30 min) following MA. K and α angle are also used to quantify the dynamics of clot formation. Reproduced from [12].

For TEG^®^ 5000, the blood sample is placed in a heated cup and the cup is then oscillated through a 4°45′ rotation (Figure 5). A pin is suspended within the cup by a torsion wire. Before clotting, the shear that develops between the cup and pin results in a viscous shear of the test sample and therefore, no movement of the pin. As clot strength develops, the fluid gradually develops an elastic element, which results in movement of the pin. Instead of the light-based system used by Dr. Hartert, the TEG^®^ 5000 uses an electromechanical proximity sensor to detect rotation of the pin. The total deflection of the pin is tracked and can be plotted as shown in Figure 6. A range of assays have been developed for the TEG^®^ 5000 system, which includes those shown in Table 1.

### 4.2. ROTEM^®^ Delta (Tem International GmbH)

The ROTEM^®^ delta system was developed after the TEG^®^ 5000 system and utilizes a similar approach with cups and pins making up the heart of the instrument. The difference lies in the actuation and detection of the cup and pin assembly. The cup is held stationary and heated, and a rotational force is applied to the pin. If there is no resistance to pin motion, the pin will rotate a full 4°75′. As the clot builds up, it restricts the rotation of the pin. The restriction of pin rotation is inversely proportional to the clot strength. Rotation of the pin is measured in a manner similar to that originally developed by Hartert; a collimated light beam from a light emitting diode is reflected off a mirror coupled to the pin. The motion of the pin is then tracked by a photosensor. As with the TEG^®^ 5000, the ROTEM^®^ delta system has a long clinical history. Various applications have been widely published [13,14] and a wide range of assays have been developed, including those shown below (Table 2).

### 4.3. Sonoclot^®^ (Sienco)

**Figure 7:**
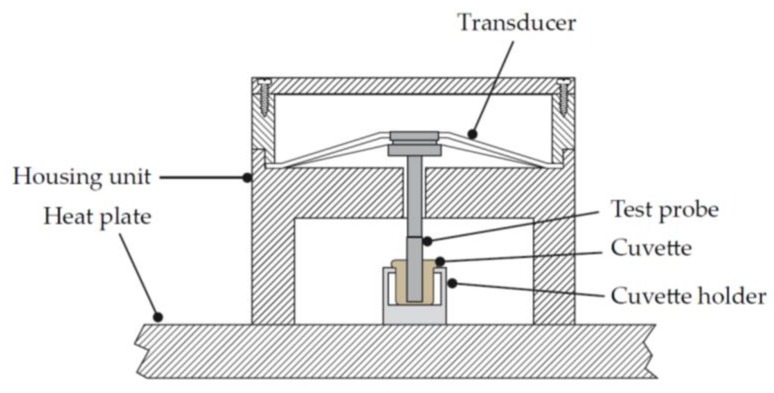
Schematic of the Sonoclot^®^ system. The sample to be tested is placed in a cuvette within the housing unit. The linear test probe is then lowered into the cuvette and is in contact with the sample to be tested. An electric signal through the transducer produces a linear oscillatory motion of the test probe whilst a heat plate warms the sample to be tested through the cuvette holder. Adapted from US Patent 5,138,872 [16].

Sonoclot^®^ is another legacy device developed by Sienco. The Sonoclot^®^ device differs from other legacy systems in that it is not a rotational-based system, but a linear motion system (Figure 7). A hollow, open-ended plastic probe is connected to the transducer head and immersed in a sample containing different coagulation activators or inhibitors, depending on the assay [15]. The probe oscillates vertically within the sample, with the oscillating motion modified as the blood sample begins to clot. The transducer outputs a signal that is proportional to the deflection of the test probe. The changes in the oscillatory pattern of the test probe are directly correlated to the viscoelastic properties of the sample. Measurements are taken over time and plotted along a time axis, similar to other legacy systems. The newest generation of the Sonoclot Analyzer is fully digitalized; however, it is not yet approved by the American Food and Drug Administration (FDA) and therefore is only utilized outside of the US. 

## 5. Towards Site of Care

The last two decades have seen rapid expansion of the use of viscoelastic hemostatic assays in an ever-expanding range of clinical applications. The devices described above did much to lay the groundwork for the clinical relevance of viscoelastic hemostatic assays. They offered a holistic view of the clotting process that simply was not available with traditional assay portfolios. However, the manual nature of performing the tests has limited the growth of its clinical utilization.

All of the first-generation devices require manual pipetting of blood samples and reagents. The open-nature of the systems limits their use in point-of-care applications. For many of the potential applications of this technology (cardiac surgery, trauma, etc.) speed and proximity to the patient are critical for success [17]. To overcome these limitations, the historic players in this market have developed next-generation systems. In addition, there are new entrants to this field with new technologies.

These next-generation technologies need to target several key factors to overcome the shortcomings of their predecessors.

- **Ease of use**—The ideal systems should decrease the overall number of manual transfer steps, or eliminate them altogether. Where possible, reagents should be dosed and reconstituted automatically. The user interface should be intuitive, with as little intervention as possible as the target users for these devices will extend beyond skilled medical laboratory technicians. Ideally, the systems would meet the requirements to be considered waived under Clinical Laboratory Improvement Amendments (CLIAs).
- **Size**—In order to optimize their use, the devices should be able to be utilized very close to the patient. This means they need to take up little valuable space in already crowded emergency rooms, cath labs and operating theatres. Taking up as little of this valuable real estate as possible is critical.
- **Resistance to ambient conditions**—Having a clear view of a patient’s overall hemostasis as early as possible can be a lifesaving benefit in certain emergencies. In clinical scenarios such as stroke or massive trauma, getting feedback on the underlying hemostatic status of a patient as early as possible is critical. For this reason, next-generation devices may be used outside of traditional clinical settings. Environments such as ambulances, helicopters or even the battlefield should be considered. Fundamental technologies that may be sensitive to vibration or temperature may not be suitable for certain applications.
- **Remote viewing**—Fundamentally, these assays produce a sequence of results over a period of time and allow the entire clinical team to view results as they’re being developed is important to timely intervention. Having the capability for readings to be displayed in real-time in the operating room may be critical. Allowing emergency department physicians to watch results develop whilst patients are being transported to the hospital will help prepare them for the arival of that patient.

Furthermore, unlike many of the first-generation devices that had significant inter-laboratory variance, with coefficients of variation >10% [18,19,20], these newer-generation devices have demonstrated reduced variance [21]. Whilst older-generation devices enabled users to focus on running channels of interest, newer devices detailed below require users to run entire cartridges. While this makes the assay process more streamlined and precise, it may be associated with increased cost.

### 5.1. TEG^®^ 6s (Haemonetics)

To overcome the issues inherent with a manual device, Haemonetics released the TEG^®^ 6s. The TEG^®^ 6s is a cartridge-based system that automates all sample aliquoting, reagent mixing and testing. The system consists of a pneumatically controlled microfluidic cartridge that takes an initial blood sample and divides it across four distinct test channels. Each test channel includes a set of reagents to conduct four different assays simultaneously.

**Figure 8:**
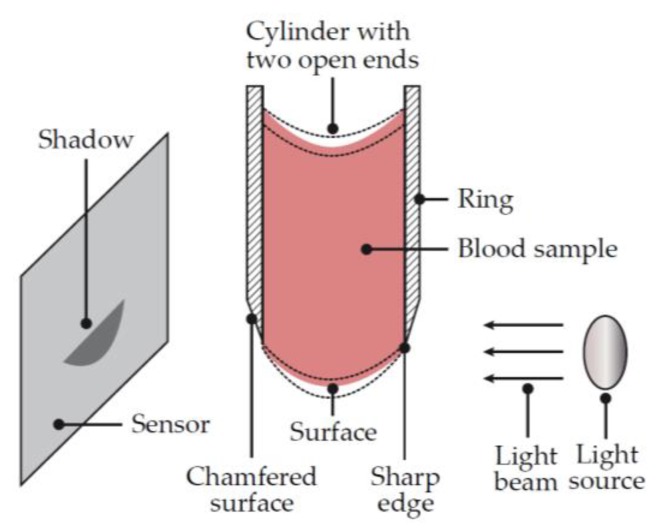
Schematic representation of the TEG^®^ 6s. The diameter of the ring and cylinder is sized to allow the blood sample to be held within the cylinder by surface tension, without support at the bottom surface. The light source (light emitting diode) directs a beam of light at the blood sample, casting a shadow on the sensor. As the surface of the sample is excited by the light beam, the sample oscillates and the corresponding shadow becomes larger or smaller depending upon the elasticity of the blood sample. The resonant frequency of the blood sample is determined before, during and after coagulation. Changes in the resonant frequency of the sample are indicative of the hemostasis characteristics of the blood sample. Adapted from US Patent 7,879,615 B2 [24].

In order to bring the test methodology to a cartridge-based system, Haemonetics developed a new measurement technique. A fluid’s shear modulus is proportional to its resonant frequency; the frequency at which a material will resonate when exposed to an excitation. The testing mechanism of the TEG^®^ 6s consists of a droplet of fluid suspended between a light source and a photodetector (Figure 8). The droplet of fluid is excited by the displacement of a piezoelectric actuator. The piezoelectric actuator is driven with a function that includes all frequencies between 25 and 400 Hz, at increments of 0.25 Hz. When subjected to this range of frequencies, the sample will resonate at its resonant frequency. The optical sensors measure the displacement of the droplet and calculate the frequency of vibration with a Fourier transform.

The TEG^®^ 6s system has gained regulatory clearance in the USA and Europe amongst other international markets and has been compared to first-generation thromboelastography systems in the literature [19,21,22,23]. The assays available with the TEG^®^ 6s are similar to the TEG^®^ 5000 and are available in three cartridge configurations. The Global Hemostasis cartridge includes Kaolin TEG^®^ with Heparinase, RapidTEG™ and Functional Fibrinogen. It is also possible to have the Global Hemostasis cartridge without KaolinTEG^®^ with Heparinase. The Platelet Mapping cartridge adds channels with adenosine diphosphate and arachidonic acid reagents to activate specific platelet activation pathways.

### 5.2. ROTEM^®^ Sigma (Instrumentation Laboratories)

The ROTEM^®^ sigma system is a transfer of the manual ROTEM^®^ delta system to a cartridge-based system, which maintains the cup and pin methodology. The pins are embedded within the cartridge and actuated by the device when installed. The cartridge is capable of withdrawing a sample of blood from a tube and aliquoting it across four channels simultaneously. Fluid flow is controlled, and reagents are reconstituted automatically. The appropriate sample volume is deposited into each cup and the tests are initiated.

The ROTEM^®^ sigma system offers two cartridges, the Sigma Complete and the Sigma Complete with Heparinase. Each cartridge provides the FIBTEM^®^, EXTEM^®^, INTEM^®^ and APTEM^®^ tests. The Heparinase cartridge allows for the neutralization of Heparin. The Sigma system has CE marking (Conformité Européene) for use in Europe, has been widely published, and been compared to the previous generation of thromboelastometry devices [23,25,26].

### 5.3. Quantra^®^ (HemoSonics)

The Quantra^®^ system developed by HemoSonics, which is CE marked and FDA cleared, utilizes Sonic Estimation of Elasticity via Resonance (SEER) technology [27]. This basic methodology has been used in the past in order to diagnose in vivo clots such as in deep vein thrombosis. Most diagnostic ultrasound devices use the echogenicity of a material in order to estimate its density. SEER technology analyzes the waveforms of the material and estimates the modulus of that material. By doing so, one can estimate if a clot has formed inside a vessel, and the extent of that clot. HemoSonics adapted this technology and refined it to get a more accurate estimate of the modulus of a fluid within a test chamber. The SEER technique has three distinct steps. The first step is data acquisition. This phase is broken up into a number of substeps. First, a low energy sensing pulse is sent into the test chamber. This sensing pulse provides a map of the location of acoustic scatterers within the sample. In the case of whole blood, the primary acoustic scatterers are red blood cells. Next, a higher energy forcing pulse is sent into the test chamber. This forcing pulse results in a displacement of the material within the test chamber. What follows is a series of sensing pulses to provide more maps of the acoustic scatterers. The next phase of the test is motion estimation. In order to estimate motion, a Finite Difference Time Domain model is utilized. This model outputs the relative motion of the acoustic scatterers. The output of this second phase is displacement vs. time waveform. The third phase of the test is to analyze the time displacement waveform for frequency. From this evaluation, the modulus of the fluid in the test chamber is estimated.

## 6. Emerging Technologies

Several emerging technologies are currently in development for point-of-care hemostatic testing, including microfluidics, fluorescent microscopy, electrochemical sensing, photoacoustic detection, and micro/nano electromechanical systems (MEMS/NEMS) [28]. In the section below, we outline several new devices/technologies that are currently under investigation for use as viscoelastic hemostatic assays; however, these technologies may also have other applications, such as monitoring microfluid dynamics and shear force in coagulation [29,30,31,32,33,34].

### 6.1. Laser Speckle Rheometry (Massachusettes General Hospital)

**Figure 9:**
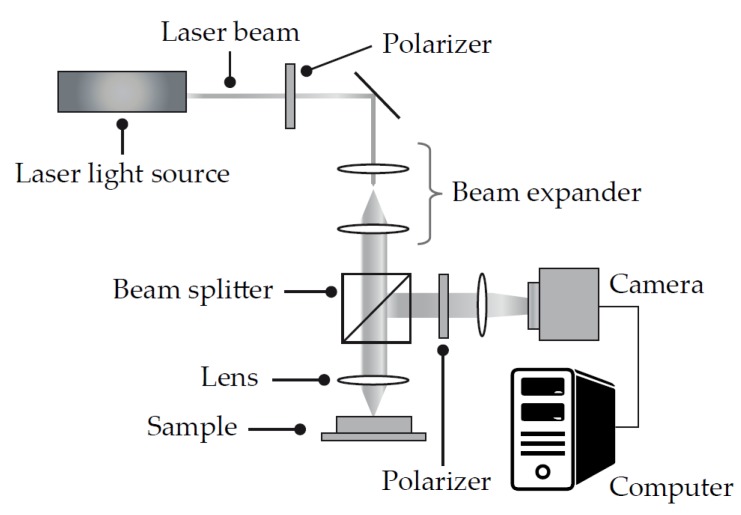
Schematic representation of the Laser Speckle Rheometry system. A laser light source shines a laser beam through a beam splitter onto a sample. The speckle pattern is detected by a camera and processed as described above. By analyzing the laser speckle pattern, the system can estimate the Brownian motion of the material. Brownian motion in such a sample is directly related to the viscoelastic properties of the material. During the blood clotting process, the change in viscoelastic properties can be evaluated and strongly correlated to parameters found in predicate viscoelastic hemostasis analyzers. Adapted from US Patent 8,772,039 B2 [35].

Laser Speckle Rheometry (Figure 9) is a technology being pioneered at the Wellman Center for Photomedicine at Massachusetts General Hospital and Harvard Medical School [35,36]. The technology has applications in a range of areas, including the catheter-based assessment of atherosclerotic plaques. One of the leading applications for the technology is as a viscoelastic hemostatic assay.

Laser speckle is a random intensity pattern that occurs by the interference of coherent light scattered from tissue. The technology uses a camera-based data acquisition system that detects the speckle pattern reflected from a material. A software algorithm detects changes in the speckle pattern over time. These changes are very sensitive to the passive Brownian motion of light scattering particles.

### 6.2. Mechanical Resonant Frequency (Abram Scientific)

Abram Scientific is developing a small, handheld approach to viscoelastic hemostatic monitoring [37]. The core technology is based on the concept that the resonant frequency of an object is affected by the medium that is surrounding that object. This can be described as viscoelastic damping. The measurement mechanism consists of a small vibrating element that is surrounded by the test sample. The vibrating element is actuated via an electric signal with a swept frequency. There is a detection mechanism that detects the resulting vibration frequency of the element. As the properties of the sample surrounding the vibrating element change, the frequency of vibration changes. In this way, the system is capable of detecting the viscoelastic properties of the sample. The Abrams Scientific device has the potential to be very compact with the vibrating element of the disposable device integrated into a layered microfluidic chip.

### 6.3. Ultrasonic Deformation (Levisonics)

**Figure 10:**
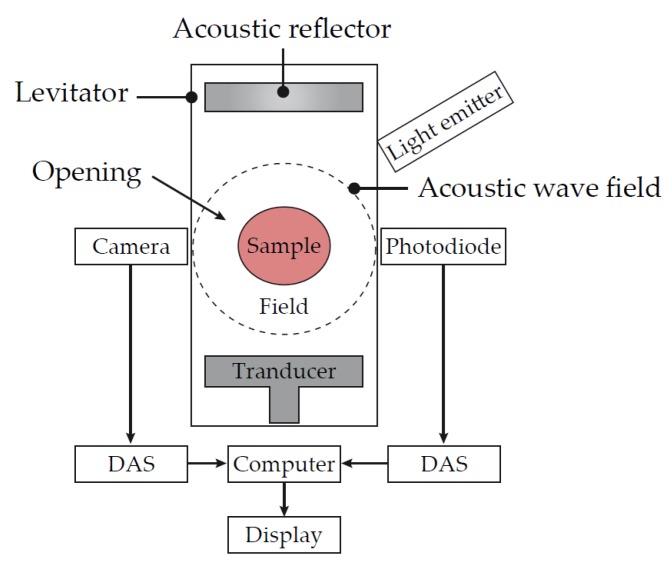
Schematic of the Levisonics ultrasonic transducer. The extent of shape deformations in the suspended blood droplet are directly correlated to the viscoelastic properties of the sample. As the amplitude of the standing wave is modulated, the shape of the sample will change from spherical to oblong. The camera system will track these shape changes and an algorithm converts them to viscoelastic properties. DAS = data acquisition system. Adapted from US Patent application 2017/0016878 A1 [38].

Levisonics was formed with technology originally developed at Boston University and Tulane University [38]. This technology consists of an ultrasonic transducer that is positioned opposite a reflector. A standing ultrasonic wave is generated between the transducer and reflector. The radiation pressure generated is sufficient to balance the gravitational force on the sample, levitating it in position between a camera and light source. While the drop of blood is positioned at the pressure node of the standing wave, the amplitude can be modulated, which produces static or oscillatory shape deformations in the sample. These shape deformations are then recorded with a high-resolution digital camera or by laser scattering detection (Figure 10).

One potentially differentiating feature of this technology is the fact that the blood sample is able to be tested whilst not in contact with any artificial surfaces. This provides two potential benefits. First, there is no disruption to the fluid sample during the clotting process. This may eliminate any mechanical shear that could affect how the clot develops. Second, the absence of artificial surfaces being exposed to the sample during the clotting process may provide a more physiological environment.

### 6.4. Parallel Plate Viscometry (Entegrion)

**Figure 11:**
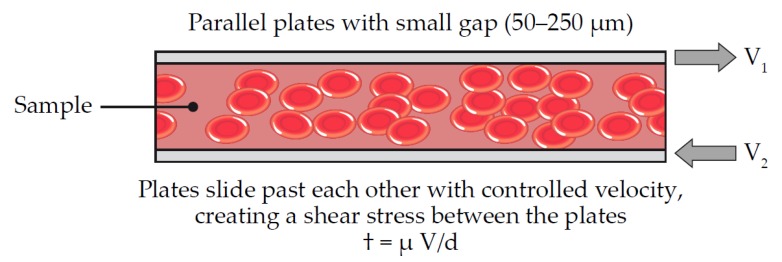
Parallel plate viscometry. Parallel plates slide past each other with controlled velocity to create a shear stress between the plates, which is represented as † = µV/d, where † = shear stress; µ = viscosity, V = V_1_ − V_2__,_ (relative linear velocity of the plates); d = gap between plates. Adapted from US Patent 8,450,078 B2 [39].

Entegrion has developed a system that closely mimics traditional rheological test platforms used outside the medical industry [39]. In essence, they have developed a parallel plate viscometer (Figure 11). Their test cartridge consists of two parallel plates, each connected to a voice coil actuator. The sample to be tested lies between the two plates. Driving signals of various amplitudes and frequencies can elicit motion between the two plates. An optical system then detects the resulting motion on the sample. All the rheological information required to calculate the viscoelastic properties of the sample can be generated with this configuration. Entegrion has been able to integrate this technology into a small, point-of-care system. The resulting small footprint allows for applications in a range of clinical settings including pre-hospital emergency medicine. 

### 6.5. Traditional Viscoelastic Testing with “Active Tips” (Enicor)

**Figure 12:**
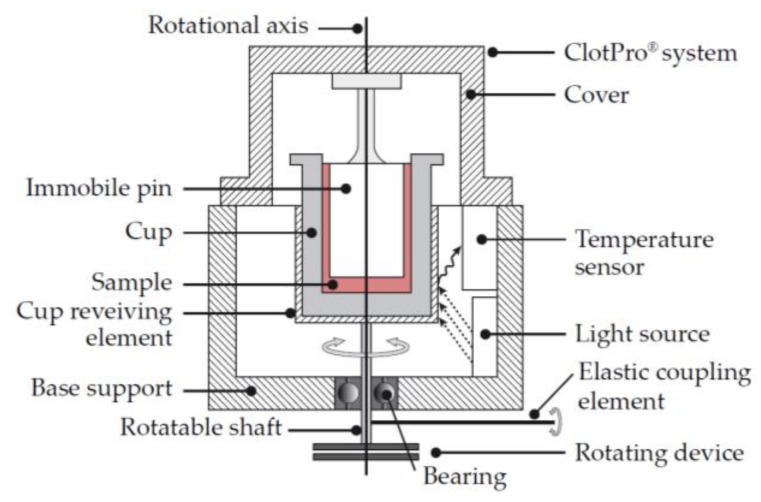
Schematic of the ClotPro^®^ system. The ClotPro^®^ system consists of a cup that contains the blood sample and a static pin, which is fixed to the cover. The cup rotates around the vertical axis, driven by an elastic coupling element, such as a spring wire, which is attached to the shaft. The light source (wavelength 1–3 µm) is placed within 75 mm of the shaft and cup-receiving element to act as a temperature control device. Adapted from International Patent WO,2018/137766,A1 [40].

Enicor has developed a novel solution for those who prefer the cost effectiveness and flexibility of an open system [40]. The measurement technology is very similar to the legacy devices such as TEG^®^ and ROTEM^®^. With TEG^®^, the cup rotates and the resulting rotation on the pin is measured. With ROTEM^®^, the cup is held stationary and a rotational force is applied to the pin, which is then measured. With the ClotPro^®^ system from enicor (Figure 12), the pin is held stationary and a rotational force is applied to the cup. The resulting rotation on the cup is measured.

With these legacy systems, multiple pipetting steps are required to meter and deliver the correct concentration of reagent into the sample cup. ClotPro^®^ has simplified this by utilizing “Active Tips”. Active tips are standard pipette tips that already contain the necessary amount of reagent. Blood is drawn into the tip and the reagent is automatically reconstituted in the correct dose. The blood is then dispensed into the cup and the test is initiated. The ClotPro^®^ system also has a test menu similar to that of ROTEM^®^.

## 7. Conclusions

The field of viscoelastic hemostatic assays is rapidly growing and generating significant clinical studies, academic research, and industry funding. Whilst older devices have been available for several decades, new technologies are being introduced that promise to make viscoelastic testing more readily available in a wider range of clinical environments. New assays are being developed to give clinicians information about their patients’ hemostasis that have never been available before. New indications that have been recently approved, or are currently under investigation, include trauma, interventional cardiology, extracorporeal cardiac assist, obstetrics and anti-coagulation therapy monitoring. The ability of viscoelastic hemostatic assays to deliver clear and readily actionable information about a patient’s overall hemostatic status will continue to improve patient care in the years ahead; however, newer technologies and devices will have to demonstrate their clinical effectiveness.

## Figures and Tables

**Table 1:** Assays developed for the TEG^®^ 5000 system.

TEG^®^ Test	Description
Kaolin TEG^®^	Intrinsic pathway-activated assay.
Kaolin TEG^®^ with Heparinase	Eliminates the effect of heparin. Used in conjunction with Kaolin TEG^®^ to assess heparin effect.
RapidTEG^TM^	Extrinsic and Intrinsic pathways to speed coagulation process and rapidly assess coagulation properties.
TEG^®^ Functional Fibrinogen	Extrinsic pathway with platelet inhibitor to restrict platelet function. Allows for quantification of fibrinogen contribution to clot strength.
TEG^®^ Platelet Mapping^TM^	Utilises a platelet receptor-specific tracing in conjunction with Kaolin TEG^®^. Identifies level of platelet inhibition and aggregation.

**Table 2:** Assays developed for the ROTEM^®^ delta system.

ROTEM^®^ Test	Description
INTEM^®^	Contact activation with phospholipid and ellagic acid.
EXTEM^®^	Tissue factor activation.
HEPTEM^®^	Heparinase to neutralize heparin. Used in conjunction with INTEM^®^ to assess heparin effect.
APTEM^®^	Inhibits fibrinolysis. Used in conjunction with EXTEM^®^ to assess fibrinolysis.
FIBTEM^®^	Blocks platelet contribution to clot formation. Allows for quantification of fibrinogen contribution to clot strength.
